# Cyanogenesis of Wild Lima Bean (*Phaseolus lunatus* L.) Is an Efficient Direct Defence in Nature

**DOI:** 10.1371/journal.pone.0005450

**Published:** 2009-05-08

**Authors:** Daniel J. Ballhorn, Stefanie Kautz, Martin Heil, Adrian D. Hegeman

**Affiliations:** 1 Department of General Botany – Plant Ecology, Universität Duisburg-Essen, Essen, Germany; 2 Departamento de Ingeniería Genética, CINVESTAV – Irapuato, Guanajuato, México; 3 Department of Horticultural Science, University of Minnesota, St. Paul, Minnesota, United States of America; CNRS UMR 8079/Université Paris-Sud, France

## Abstract

In natural systems plants face a plethora of antagonists and thus have evolved multiple defence strategies. Lima bean (*Phaseolus lunatus* L.) is a model plant for studies of inducible indirect anti-herbivore defences including the production of volatile organic compounds (VOCs) and extrafloral nectar (EFN). In contrast, studies on direct chemical defence mechanisms as crucial components of lima beans' defence syndrome under natural conditions are nonexistent. In this study, we focus on the cyanogenic potential (HCNp; concentration of cyanogenic glycosides) as a crucial parameter determining lima beans' cyanogenesis, i.e. the release of toxic hydrogen cyanide from preformed precursors. Quantitative variability of cyanogenesis in a natural population of wild lima bean in Mexico was significantly correlated with missing leaf area. Since existing correlations do not by necessity mean causal associations, the function of cyanogenesis as efficient plant defence was subsequently analysed in feeding trials. We used natural chrysomelid herbivores and clonal lima beans with known cyanogenic features produced from field-grown mother plants. We show that in addition to extensively investigated indirect defences, cyanogenesis has to be considered as an important direct defensive trait affecting lima beans' overall defence in nature. Our results indicate the general importance of analysing ‘multiple defence syndromes’ rather than single defence mechanisms in future functional analyses of plant defences.

## Introduction

Lima bean (Fabaceae: *Phaseolus lunatus* L.) represents a prominent experimental model plant for studies of inducible indirect plant defences against herbivores [1]. These indirect (carnivore attracting) defences include the release of herbivore-induced volatile organic compounds (VOCs) and secretion of extrafloral nectar (EFN). Both types of indirect defences have been investigated extensively for the last decade under laboratory [2]–[8] and, more recently, natural field conditions [9]–[14]. In addition to directly attracting carnivores, VOCs of lima bean play a role in defence-associated signalling between plants [6], and as external signal for priming of induced indirect defences within a single plant [15].

In contrast to indirect defences, the efficiency of lima beans' direct chemical defences under natural conditions has not been studied until now. A characteristic direct chemical defence of lima bean is cyanogenesis, i.e. the release of toxic hydrogen cyanide (HCN) from preformed cyanide-containing compounds in response to cell damage [16]–[20]. Plants do not rely on a single defence mechanism, but rather express multiple defences comprising the constitutive and induced synthesis of many chemical compounds as well as the production of structural traits [e.g. 21]–[24]. The combination of different traits often leads to the evolution of multiple defence syndromes, since the association with specific ecological interactions results in co-variation of defensive traits [25], [26]. In a recent series of conceptual and experimental studies, the evolution of defence syndromes has been compellingly illustrated for milkweeds (*Asclepias* sp.) [27], [28]. Nevertheless, the functional interplay of different plant traits involved in a plant's overall defences under natural conditions is poorly understood in many cases. To better understand this functional interplay of different defensive traits, multiple contributing components must be analysed thoroughly.

In natural systems functional analyses of specific plant traits are generally complicated by the variability of internal and external factors affecting the trait of interest. Physiology of plants and plants' attractiveness to herbivores is known to vary widely depending on plant and leaf age [17], [29]. In addition, microclimatic conditions have a strong impact on plant characteristics and consequently on the interaction of plants with higher trophic levels [30]. Thus, to investigate the efficiency of lima beans' cyanogenesis as a direct defence in nature, we applied an integrative approach combining analyses for quantitative correlations of cyanogenic potential (HCNp; the maximum amount of cyanide that can be released by a given tissue [31]) and herbivory in the field with feeding trials under controlled conditions. In some plant species — as for example in *Sorghum bicolor* — intermediates formed in the process of cyanogenesis can be metabolized and correspondingly the amount of cyanide, which can be released from a given tissue, is lower than the amount that is accumulated in cyanogenic precursors [32]. However, in lima beans with high *β*-glucosidase activity as is often found in wildtypes, the HCNp is a good measure for cyanogenesis because the amount of cyanide contained in cyanogenic precursors corresponds closely to the total amount that is released in response to cell disintegration [17].

Negative correlations of cyanogenesis and resistance to different types of herbivores have been demonstrated convincingly for several plant species on both extensive [33] and local scales [34], [35]. Confirming these results in field studies on natural lima bean populations, we found a distinct negative correlation between leaf damage and HCNp. As existing correlations observed in nature do not necessarily mean causal relations, we conducted additional feeding trials under out-door conditions in South Mexico with two natural insect herbivores of lima bean (Chrysomelidae: *Cerotoma ruficornis* and *Gynandrobrotica guerreroensis*). Before natural variability of cyanogenesis may be used for functional analysis of its defensive efficiency in herbivore-plant interactions, the quantitative variation among cyanogenic features must be known. In the present study, we produced clonal plants by stem cuttings that were derived from mother plants growing at a natural site characterized by distinct differences in HCNp. Our experimental design thus allowed us to measure quantitative effects of HCNp on herbivores. This integrative approach considered both the efficiency of cyanogenesis as a direct defence in nature and its quantitative effects in controlled feeding trials.

## Results and Discussion

### Cyanogenic Potential of Lima Bean in Nature

**Figure 1:**
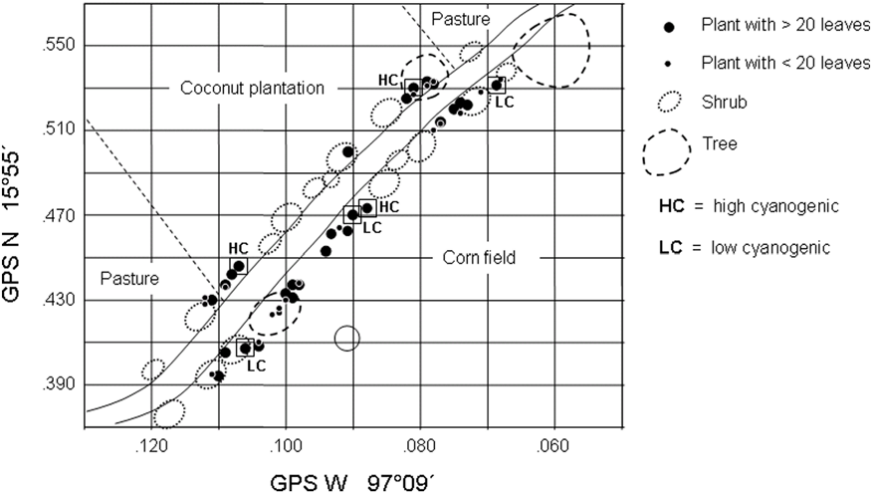
Schematic illustration of the sample collection site. Positions of individual plants at the natural site were generated with GPS-data and combined with schematic illustration of vegetation and surrounding land use pattern. Plants of different size and number of leaves were included in the analysis. Individuals in boxes (HC and LC) were selected for the production of clones for consecutive feeding trials.

**Figure 2:**
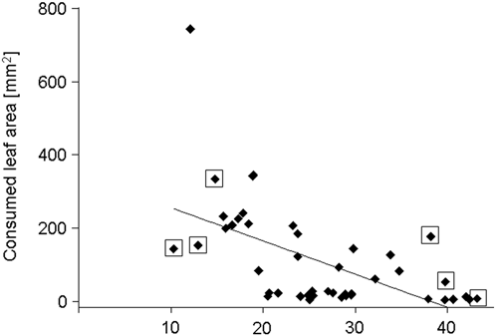
Association of missing leaf area and HCNp in nature. Individual leaves of field-grown lima bean plants (*n* = 1 leaf of a defined ontogenetic stage per plant; *n* = 46 plants) were quantitatively analysed for removed leaf area and concentration of cyanogenic precursors (HCNp). Data were analysed using Pearson's correlation (*P*<0.01). Individuals in boxes (HC and LC) were selected for the production of clones for consecutive feeding trials.

The cyanogenic potential (HCNp) of 46 individual field-grown lima bean plants was quantitatively analysed. At the natural field site, we considered leaves of a defined developmental stage (young, fully unfolded leaves inserting three positions down the apex) to avoid ontogenetic variability of plant traits including ‘Plant Size’ and ‘Light Exposure’ (Fig. 1) as random factors in an multivariate general linear model (GLM) for assessing potential effects of these factors on HCNp and consumed leaf area (Table 1). Among plants growing in nature, HCNp varied substantially ranging from 10.32 to 43.18 µmol HCN g^−1^ fwt (*n* = 46 leaves; Fig. 2). Lima beans growing in full sunlight showed no significant differences in HCNp when compared to plants growing under shaded conditions (according Mann-Whitney-U test: *Z* = −0.770, *P* = 0.441; *n* = 24 plants in full sun, and *n* = 22 growing under shaded conditions; two-tailed *P*-values are reported; Fig. 1). In addition, we found no significant differences in HCNp depending on plant size (*Z* = −0.569, *P* = 0.570; *n* = 19 plants with less than 20 leaves, and *n* = 27 plants with more than 20 leaves; Fig. 1). Consequently, the GLM predicted no effects of ‘Plant Size’ or ‘Light Exposure’ on HCNp (Table 1). These findings indicate that microclimatic conditions as well plant age or size have limited impact on leaf HCNp in lima bean plants at natural sites. Alternatively, the results of this field study suggest that the observed quantitative variability of cyanogenic plant features is under genetic control as has been observed for many other cyanogenic plant species [e.g. 30], [36]–[38]. This high degree of genetic control of cyanogenesis in wild type lima bean was confirmed in our cloning experiments where plant material with defined cyanogenic features was generated for feeding trials.

**Table 1:** Effects of plant size and light exposure on HCNp and missing leaf area.

Source		Dependent variable	*SS*	*df*	*F*	*P*
Plant Size	Hypothesis	HCNp	4.074	1	0.052	0.820
	Error		3352.939	43		
Light Exposure	Hypothesis		59.553	1	0.764	0.387
	Error		3352.939	43		
Plant Size	Hypothesis	MLA	0.258	1	0.130	0.720
	Error		85.278	43		
Light Exposure	Hypothesis		2.273	1	1.146	0.290
	Error		85.278	43		

Results obtained using the GLM (general linear model) for analysis of variance after a multivariate design with ‘HCNp’ and ‘Missing Leaf Area’ (MLA) as variables. Factors were ‘Plant Size’ (i.e. less than 20 leaves vs. 20 leaves and more) and ‘Light Exposure’ (i.e. light vs. shade).

### Herbivory at Natural Sites

Among lima bean plants in the field, herbivore damage measured as missing leaf area (of defined leaf stages) showed high quantitative variability (0–741 mm^2^ corresponding to 0–33% leaf area removed; Fig. 2). None of the leaves considered in this study was missing completely (i.e. damaged on the 100% level). Analysis of individual leaf cyanogenic features revealed a significant negative correlation of leaf damage and HCNp (according to Pearson's correlation: *r* = −0.567, *P*<0.001; *n* = 46; Fig. 2). Plants with high cyanogenic young leaves showed less damage even of older leaf stages than plants with lower cyanogenic young leaves (pers. observ.). Thus, the correlation of HCNp and missing leaf area in individual leaves was a good predictor for herbivore damage of total plants. This is in accordance with own previous laboratory analyses of HCNp ontogenetic variability in lima beans, since plants characterized by high HCNp in their young leaves showed consistently higher HCNp in intermediate and mature leaf developmental stages than plants with low HCNp in young leaves [17].

In contrast to significant effects of HCNp on leaf damage, neither plant size (*Z* = −0.312, *P* = 0.755) nor plant exposure to light (*Z* = −0.341, *P* = 0.733) significantly affected herbivores' preferences (according to Mann-Whitney-U test, two-tailed *P*-values are reported). Consequently, GLM predicted no significant effects of ‘Plant Size’ or ‘Light Exposure’ on the degree of herbivory observed (Table 1). Critical evaluation of small-scale site-specific variability in growth conditions and plant size (as we conducted here) is crucial to predict causal associations between a defensive plant trait and its efficiency in herbivore deterrence under natural conditions. Although not considered in many field studies, the high impact of positional effects (such as light exposure) as well as effects of plant size or age on the degree of herbivory observed in natural systems is well documented [39]–[42]. Thus, variability of site-specific conditions and plant morphological parameters must be included in meaningful analyses of natural plant-herbivore systems.

### Feeding Trials

Field-observations of negatively correlated herbivore damage (missing leaf area) and HCNp suggest that cyanogenesis is important in anti-herbivore defence of lima bean at natural sites, whereas plant size and light exposure of the respective plant did not significantly affect damage of defined leaves by herbivores. Despite these observations, however, a broad array of external factors including microclimatic conditions [43] and plant morphological parameters [44], biotic factors, such as aggregation phenomena of herbivores [45], sporadic appearance of carnivores [46], [47] as well as patch heterogeneity of neighbouring plants [48], [49] may strongly determine the outcome of damage to individual plants by herbivores.

In order to confirm our field observations on the efficiency of lima beans' cyanide production as plant defence against herbivores, we conducted feeding experiments with two chrysomelid herbivores (*Gynandrobrotica guerreroensis* and *Cerotoma ruficornis*). These insects commonly occurred on lima bean plants and were collected in natural sex-ratios in the field. Leaf material for the feeding trials was derived from lima bean cuttings prepared from three high (HC) and three low (LC) cyanide containing mother plants growing at the natural field site (Figs. 1 and 2). HC- and LC-cuttings from these plants were cultivated under herbivore (and carnivore) free outdoor-conditions to reduce variation of external factors [17]–[19], [50]. Quantitative analyses of HCNp in leaves of defined developmental stages using clonal plants (according to plants at natural site three insertion positions down the apex) revealed similar cyanide concentrations as compared to the mother plants and showed low variation in HCNp within HC- and LC-groups. Cyanogenic potential in leaves of clones belonging to the HC-group ranged from 26.19 to 37.24 µmol HCN g^−1^ leaf fwt whereas HCNp in plants of the LC-group was lower and ranged from 5.25 to 14.51 µmol HCN g^−1^ leaf fwt. To exclude unknown variability of HCNp from the experimental setup, we measured cyanide in remaining individual leaflets after they had been used in feeding trials. Both lateral leaflets of trifoliate leaves were used in feeding trials according to Heil [9]. Comparative analyses of both lateral leaflets of individual trifoliate leaves revealed a variance of 4.90±4.01% in HCNp (mean±s.e.m.; *n* = 84 leaves) when considering the highest cyanogenic leaflet of each pair as 100%. These leaflets with defined cyanogenic features therefore allowed for utilization of naturally occurring HCNp variability for functional analyses of its efficiency in anti-herbivore defence under experimental feeding trial conditions.

### Choice Behaviour of Beetles

**Figure 3:**
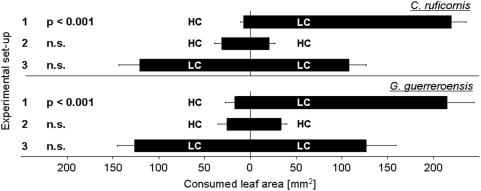
Choice behaviour of insects in feeding trials. Choice behaviour of both beetle species (*C. ruficornis* and *G. guerreroensis*) was tested in feeding trials, in which leaf material of different cyanogenic quality was offered. In trial 1 high (HC) and low cyanogenic (LC) leaf material was presented to the beetles, whereas trials 2 and 3 served as controls and the beetles were given the choice to select between leaves of similar quality (HC leaves in trial 2 and LC leaves in trial 3), respectively. Data on removed leaf area are means (±s.e.m.) of *n* = 14 replications per setup.

Cuttings produced from different mother plants were grouped (HC and LC) according to their HCNp. These groups were pooled to avoid potential effects on herbivores resulting from individually different attractiveness of plant material depending on other factors than cyanogenesis. Pairs of leaflets with defined HCNp from different individual plants were used in trials (Fig. 3). Depending on the experimental set-up, we compared high vs. low (1), high vs. high (2) and low vs. low (3) cyanogenic leaflets (Fig. 3). Both insect herbivores investigated showed significant preferences for low over high cyanogenic leaf material in feeding trials (*n* = 14 trials per setup), as measured by consumed leaf area per time [trial 1: *Z* = −3.296, *P* = 0.001 (*C. ruficornis*); *Z* = −3.296, *P* = 0.001 (*G. guerreroensis*) according to Wilcoxon signed rank test]. When given the choice between leaflets of similar HCNp, no significant preference was observed [trial 2: *Z* = −0.973, *P* = 0.331 (*C. ruficornis*); *Z* = −0.220, *P* = 0.826 (*G. guerreroensis*); trial 3: *Z* = −0.596, *P* = 0.551 (*C. ruficornis*); *Z* = −0.031, *P* = 0.975 (*G. guerreroensis*) according to Wilcoxon signed rank test].

Our results obtained under feeding trial conditions (i) demonstrate a central role of HCNp for feeding choice behaviour of both beetle species and (ii) provide a causal quantitative explanation for variability of feeding damage observed on individual lima bean plants in nature.

### Leaf Consumption and Incorporated Cyanide

**Figure 4:**
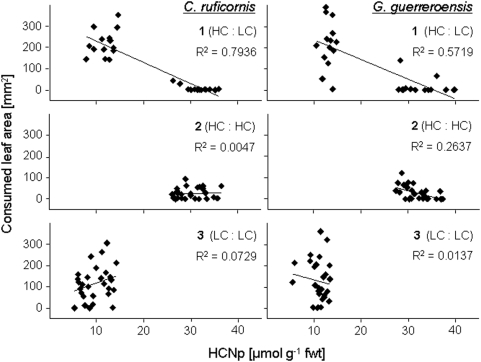
Quantitative leaf consumption vs. HCNp in feeding trials. Consumption of leaf area from leaves of different quality (HC = high cyanogenic, LC = low cyanogenic) by both beetle species under different experimental setups (trials 1, 2 and 3; *n* = 14 trials per setup) was correlated to the HCNp of the individual leaves (Pearson's correlation; *P*<0.01).

**Figure 5:**
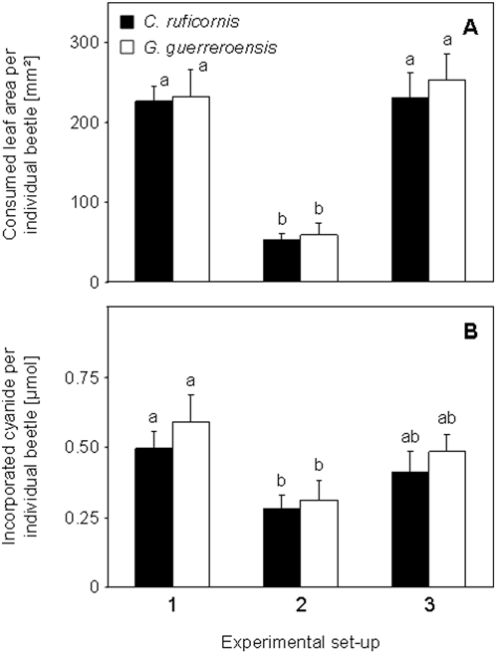
Total leaf consumption and amount of cyanide ingested in feeding trials. Included were leaves of different cyanogenic quality (HC and LC) in trial 1 or leaves of similar HCNp (HC leaves in trial 2 and LC leaves in trial 3). Data are means (±s.e.m.). Among treatments and species means of consumed leaf area marked with different letters in the upper panel are significantly different [according to *post-hoc* analysis after one-way ANOVA (LSD; *P*<0.05)]. A. Total leaf area consumed in feeding trials by both beetle species. B. Total amount of cyanide in ingested leaf material by both beetle species.

Beetles' feeding behaviour showed that the concentration of cyanide quantitatively affects repellent activity of leaves and thus represents an important measure of plant defence (Fig. 4). To address the question of whether a quantitative toxic threshold exists, we balanced the total amount of cyanide incorporated by each beetle in a respective 24 h feeding trial using missing leaf area and HCNp of leaflets offered. Individual beetles of both species consumed significantly more leaf material in trials 1 and 3 than in trial 2 (according to LSD *post hoc* analysis after one-way ANOVA: *F* = 23.046, *df* = 41, *P*<0.001, (*C. ruficornis*) and *F* = 14.002, *df* = 41, *P*<0.001, (*G. guerreroensis*) (Fig. 5A). Thus, total leaf consumption was increased when LC leaf material was available compared to setup 2, in which exclusively HC leaves were offered (Fig. 5A). After correcting for lower body weight of *C. ruficornis* [18.1±4.2 mg (*n* = 42)] as compared to *G. guerreroensis* [22.3±5.1 mg (*n* = 42)] total ingested leaf material was slightly higher for *G. guerreroensis* in all feeding trials (Fig. 5A).

Extensive consumption of LC leaf material in trails 1 and 3 resulted an higher total incorporation of cyanide as compared to amounts incorporated in HC∶HC trials (2) (Fig. 5B). ‘Setup’ was a significant source of variance [according to LSD *post hoc* analysis after one-way ANOVA: *F* = 3.253, *df* = 41, *P*<0.049, (*C. ruficornis*) and *F* = 3.039, *df* = 41, *P*<0.059, (*G. guerreroensis*)]. These results are in accordance with findings in an earlier study [17] and indicate that HC plants are significantly better defended than LC plants showing a non-linear dose-response relationship. The impact of toxic compounds generally depends on the intake per body weight and time (dose), but varies depending on the dilution with non toxic material and on the quantitative availability of plant compounds required for detoxification processes [51]–[54]. In insects – and plants – *β*-cyanoalanine synthase is an enzyme that catalyzes the conversion of cysteine and cyanide to produce *β*-cyanoalanine and sulfide. *β*-cyanoalanine can be readily metabolized into asparagine [55], [56]. Beetles can tolerate a higher dose of cyanogenic compounds when feeding on LC leaf material than HC material either because of the larger extent of dilution in LC material and/or potentially higher availability of cysteine.

While feeding trials provide striking evidence supporting the role of cyanogenesis as a causal agent moderating herbivore feeding damage in lima beans, it is interesting to see whether the results obtained in feeding trials can be corroborated in the natural system. In addition to livestock vertebrate herbivores, such as cows, goats, horses and donkeys, lima bean in South Mexico is threatened by a diverse range of natural arthropod herbivores including locusts (Pyrgomorphoidea: Pyrgomorphidae: *Sphenarium borrei* Bol. and one undetermined species belonging to the superfamily Acridoidea), caterpillars (Hesperidae: *Proteus urbanus* L., Saturniidae: *Automeris io* Fabricius and one species presumably belonging to the family Geometridae) and a species-complex of at least five sympatric chrysomelid beetles (pers. observ.). One must question whether the herbivores used in the study are of relevance in the natural system.

Chrysomelids used here represented the most prominent insect herbivores found on wild lima bean in coastal area of Oaxaca (pers. observ.). The two species (*Gynandrobrotica guerreroensis* and *Cerotoma ruficornis*), which we selected for feeding trials, were the most abundant herbivores among Chrysomelids at the study site in August 2007. Feeding damage observed in wild lima bean population could be widely assigned to *G. guerreroensis* and *C. ruficornis* by direct observation of feeding beetles and under consideration of typical feeding patterns found on leaves. Observations in nature supported the use of *G. guerreroensis* and *C. ruficornis* as representative herbivores in feeding trials and strengthen the significance of the results with relevance to the situation at field sites.

### Conclusion

In our present study, we demonstrated that cyanogenesis of lima bean represents an efficient defence against insect herbivores in nature. Quantitative correlations of herbivore damage and cyanogenesis in leaves observed in a natural population of lima bean in Mexico were confirmed under experimental conditions. Balancing leaf consumption and cyanide intake by two natural herbivores revealed a non linear dose-response relationship further indicating substantial repellent activity of high cyanogenic plant individuals or plant parts. Results of the present study, together with recent findings on distinct trade-offs between direct (cyanogenesis) and indirect defences (VOCs) of lima bean, strongly suggest implementation of cyanogenic features in further studies on lima beans' overall defence in nature. In contrast to previous studies, which have focussed exclusively on indirect defences, our findings indicate the general need for consideration of multiple defence syndromes on plant defences rather than continuing to restrict analyses to single defence mechanisms. While the subject of indirect defence efficiency appears well documented, subsequent investigations need to apply an integrative approach and revisit the topic considering quantitative variability of cyanogenesis in natural lima bean populations.

## Materials and Methods

### Study site

Analyses were conducted in August 2007 in the coastal area of Oaxaca, Mexico. We used plant material from a wild lima bean population ca. 10 km west of Puerto Escondido (15°55′N and 097°09′W, elevation 15 m). Plants were collected along a path, which was surrounded by extensively used agricultural areas (Fig. 1). Lima bean plants at the field site were in part shaded by trees and shrubs, while other plants were in full sun. All plants had developed 11–42 leaves at the time of sampling.

### Sampling of Plant Material

Leaves of a defined ontogenetic stage were assessed for leaf damage by herbivores. These leaves inserted three positions down the apex and were fully unfolded. Use of defined developmental stages reduced variability of leaf texture due to ontogenetic characteristics. Thus, all leaves analysed here showed the same soft leaf texture. One leaf per plant individual was used (*n* = 46 plants). Leaves were cut off with a razor blade and immediately placed into Ziploc® bags (Toppits, Minden, Germany) containing moist filter paper. In the field, bags were stored in an insulation box, cooled with ice during sampling, and transported to the field laboratory for analyses.

### Quantification of Leaf Damage

In the laboratory, leaves were spread on a scaled paper and then digitally photographed (Camedia C-4000 Zoom, Olympus, Hamburg, Germany). Missing leaf area of individual leaves was quantified using the AnalySIS® software (Olympus Soft Imaging Solutions GmbH, Münster, Germany) back in Germany.

### Cyanogenic Precursor Concentration

We analysed concentration of cyanogenic precursors in leaf material, i.e. the cyanogenic potential (HCNp) according Ballhorn *et al.* [17]. The method is based on complete enzymatic degradation of cyanogenic glycosides in closed Thunberg vessels and subsequent spectrophotometric measurement (585 nm) of HCN released from the cyanide-containing compounds using the Spectroquant® cyanide test (Merck KGaA, Darmstadt, Germany). For enzymatic degradation, we used specific *β*-glucosidase isolated from rubber tree (Euphorbiaceae: *Hevea brasiliensis*). This plant species possesses the same cyanogenic glycosides as lima bean, i.e. linamarin and lotaustralin. We added external *β*-glucosidase in excess to leaf extracts to guarantee total conversion of cyanogenic glycosides into free cyanide and to accelerate the enzymatic reaction.

### Cultivation of Plants

Clonal plants used in feeding trials were propagated from stem cuttings of field-grown mother plants each containing one leaf (*n* = 18 cuttings per plant). We selected three mother plants each for propagation of high (HC; more than 25 µmol HCN g^−1^ fwt) and low (LC; less than 15 µmol HCN g^−1^ fwt) HCNp to reduce variability of cyanogenesis in the set of experimental plants. The HCNp of clonal plants quantitatively resembled the HCNp of mother plants. Cuttings were rooted in continuously moist soil from the natural site mixed 1∶1 with sand and were transferred in pure natural soil after the first roots had started to grow and then were cultivated in 250 ml plastic pots. Plants were exposed to natural conditions, watered twice a day and fertilized once a week with 50 ml of nitrogen-phosphate fertilizer (Blaukorn®-Nitrophoska®-Perfekt, Compo GmbH & Co. KG, Münster, Germany) in a concentration of 0.25 mg L^−1^. Plants were checked three to four times a day for the infestation by herbivores. Herbivores rarely appeared under controlled outdoor conditions, and those that did appear were removed immediately by hand.

### Herbivores

In feeding trials, we used phyllophagous beetles *Cerotoma ruficornis* Olivier and *Gynandrobrotica guerreroensis* Jacoby (Chrysomelidae: Galerucinae: Luperini: Subtribe Diabroticina). Chysomelids were determined by Astrid Eben (Instituto de Ecología, Veracruz, Mexico). Both beetle species have repeatedly been used in earlier field studies addressing lima bean indirect defences in nature [9], [10], [12], [15]. Beetles were collected in August 2007 on lima beans at the same study site, which was selected for assessing herbivory and parallel quantification of accumulated cyanide in leaves. Beetles were present all day long exhibiting two peaks of feeding and moving activity in the first hours after dawn (8:00 AM–10:00 AM) and dusk (7:00 PM–11:00 PM), respectively (pers. observ.). Beetles appearing on lima bean were collected representing natural ratios of sexes and ages that might display different choice behaviour [e.g. 57]. Insects were kept in transparent 250 ml plastic cups with water supplied on cotton and were deprived of food for 1 d prior to the experiment.

### Feeding Trials

Feeding experiments were carried out under controlled outdoor conditions. At the time of the experiments, potted plants were 60–80 cm tall and had developed 10–15 leaves. As for leaves quantitatively analysed for HCNp and herbivory in nature, leaves that inserted three positions down the apex were analysed for HCNp and used in feeding trials. Leaves (terminal leaflets) were quantitatively analysed for their HCNp prior to the experiment to confirm quantitative stability of cyanogenesis. All three leaflets showed high homogeneity in HCNp per trifoliate leaf excluding any position effects. In accordance to mother plants, leaves with an HCNp of more than 25.0 µmol HCN per g leaf fresh weight were classified as high cyanogenic, while leaves with less than 15.0 HCN per g leaf fresh weight were considered as low cyanogenic. In the feeding experiments, two lateral leaflets derived from leaves of different plant individuals were tested against each other. We used three different experimental setups: (1) high vs. low, (2) high vs. high, and (3) low vs. low cyanogenic leaflets. After the feeding trials, remaining leaf material of individual leaflets was weighted and analysed for HCNp.

For feeding trials, single beetles were placed in 250 ml plastic cups sealed with fabric (anti-aphid net). Experiments were run over 24 hrs and then leaflets were digitally photographed on a scale for quantification of missing leaf area using AnalySIS® software back in Germany. The two leaflets exposed to the same beetle were used as a pair for data evaluation.
